# Frequency and diversity of trypanosomes in sheep and goats from Mongo County in South Gabon, Central Africa

**DOI:** 10.14202/vetworld.2020.2502-2507

**Published:** 2020-11-24

**Authors:** Gael Darren Maganga, Larson Boundenga, Emmanuella Jacqueline Ologui-Minkue-Edzo, Linda Bohou Kombila, Telstar Ghestin Ndong Mebaley, Brice Kumulungui, Jacques François Mavoungou

**Affiliations:** 1 Centre International de Recherches Médicales de Franceville, BP 769, Franceville, Gabon; 2 Département de Zootechnologie, Institut National Supérieur d’Agronomie et de Biotechnologies, BP 901, Franceville, Gabon; 3 Department of Zootechnology, Institut de Recherche Agronomique et Forestière (IRAF-CENAREST), BP: 13354, Libreville, Gabon

**Keywords:** diversity, Gabon, goat, polymerase chain reaction, Sheep, *Trypanosoma*

## Abstract

**Background and Aim:**

Trypanosomosis is a major impediment to livestock farming in sub-Saharan Africa. It is a vector-borne disease caused by several species of protozoan parasites, namely, trypanosomes. The present study aimed to identify the diversity of trypanosome species infecting sheep and goats from Mongo County and to determine the frequency of these parasites.

**Materials and Methods:**

This study was conducted on 286 trypanotolerant goats and sheep from Mongo regions located in South Gabon, using polymerase chain reaction.

**Results:**

Analyses showed that the overall occurrence of trypanosomosis in small ruminants was 13.6% (39/286). Our results also showed that two factors, species and season, could affect the occurrence rate of *Trypanosoma*. A total of six *Trypanosoma* species were identified, two in sheep (*Trypanosoma simiae* and *Trypanosoma theileri*) and five in goats (*Trypanosoma vivax*, *T. simiae*, *T. simiae Tsavo*, *Trypanosoma congolense*, and *Trypanosoma brucei*), though *Trypanosoma simiae* was the most important species. Mixed infections were also found in goats (54.5%) and sheep (3.57%).

**Conclusion:**

Our study demonstrated that small ruminants could represent a reservoir of biodiversity for *Trypanosoma* parasites.

## Introduction

In sub-Saharan Africa, the population of small ruminants is estimated at 120 million [1]. In 2009, the total number of sheep and goats in Gabon was estimated at 222,000 [2], with approximately 143,630 raised using extensive rearing. This breeding method, which is considered economical for the breeder, forces the animal to be exposed to various pathologies, including abortive diseases [3]. Many pathogens can be the cause of abortion in small ruminants, including viruses, bacteria, fungi, and parasites [4] such as *Toxoplasma gondii*, *Neospora caninum*, and *Trypanosoma* spp. [5].

Trypanosomiasis is a vector-borne disease caused by several species of protozoan parasites, called trypanosomes [6]. African animal trypanosomiasis (AAT), also known as Nagana, is a disease that affects several mammal species, including 100 million small ruminants, with considerable annual losses of meat, estimated at about 5 billion US dollars [7,8]. It is mainly caused by the parasites *Trypanosoma congolense* and *Trypanosoma vivax* and to a lesser extent *Trypanosoma brucei brucei* [9]. In addition to the trypanosomes mentioned above, *Trypanosoma simiae*, *T. simiae* Tsavo, and *Trypanosoma theileri* have also been identified in mammals. The first two are rarely present in ruminants but are more recognized for their pathogenicity in domestic and wild pigs [10,11]. *T. theileri* is widely observed in domestic cattle [12] and wild buffalo [13]. In general, according to Mattioli *et al*. [14], wild animals are a reservoir of trypanosomes and their role in domestic animal trypanosomosis varies according to animal species, *Glossina* and the trypanosome involved. Many small ruminant herds are present in the villages of Mongo County, located in the province of Nyanga, in South Gabon. In the same region, the presence of trypanosomes has already been demonstrated in cattle herds of the SIAT ranch [15]. To date, no data on trypanosome infections are available on small ruminants in Gabon. Therefore, it is important to identify the diversity of trypanosome species infecting sheep and goats.

The present study aimed to identify the diversity of trypanosome species infecting sheep and goats from Mongo County and to determine the frequency of these parasites.

## Materials and Methods

### Ethical approval

For this study, oral consent was obtained from all breeders of small ruminants for blood sampling. Animal handling and blood sampling by the CIRMF veterinarians complied with the criteria for animal well-being.

### Study area and samples collection

The study was conducted from June 2014 to September 2014, during the dry season, and in March 2018, the rainy season, in Mongo County, located about 65 km from Tchibanga, capital of the Nyanga Province of Southwest Gabon [16]. This difference in sampling time between the rainy and dry seasons was due to the difficulty of access in this area during the rainy season.

Blood samples were collected from sheep and goats of nine villages, with at least 20 individuals per village. A total of 286 blood samples (146 sheep and 140 goats) were randomly collected. Five milliliters of whole blood were collected from the jugular vein of each animal, using a 0.8×2.5 mm Precision Glide BD Vacutainer needle and placed in EDTA tubes. Samples were then stored at −80°C.

Parameters such as host species (goats/sheep), sex (male/female), age (young/adult), and sampling season were documented during the collection. All samples were collected during the dry and rainy seasons.

### DNA extraction and polymerase chain reaction (PCR)

DNA was extracted from 200 μL of whole blood on a BioRobot EZ1 Advanced XL automat (Qiagen, Germany), using the EZ1 Virus Mini Kit version 2.0 (Qiagen), according to the manufacturer’s instructions. The DNA obtained was quantified on a Qubit (Thermo Fisher Scientific, France), using the Qubit Kit dsDNA HS Assay kit (Invitrogen, France).

The trypanosome search was done in two steps using the recombinant Taq DNA polymerase kit (Life Technologies, France). The first PCR used the primers IST1, to amplify partial region 18S and IST2, to amplify regions 5.8S and 28S, of trypanosomes [11]. The amplification is carried out with a final volume of 25 μL, containing 5 μL of DNA, 2.5 μL of 10×PCR, 2 μL of dNTPs (10 mM each), 2 μL of MgCl_2_ (50 μM), 0.24 μL of each primer (10 μM), 3 μL of Taq polymerase, and 12.72 μL of RNAse-free water (Invitrogen).

Amplification involved 7 min at 95°C, followed by 35 cycles of 1 min at 94°C, 1 min at 56°C, and 2 min at 72°C, followed by a final elongation at 72°C for 10 min.

The nested PCR used primers ITS3 and ITS4 [11]. The amplification was carried out with a final volume of 25 μL, containing 1 μL of DNA from the first PCR, 2.5 μL of 10× PCR, 2 μL of dNTP (10 mM), 2 μL of MgCl_2_ (50 mM), 0.24 μL of each primer at 10 μM, 0.3 μL of Taq polymerase, and 16.72 μL of RNAse-free water (Invitrogen). The nested PCR products were then separated using 1.5% agarose gel electrophoresis.

### Statistical analyses

Analyses were performed using R commander software. Pearson’s Chi-square (χ^2^) test was used to compare the proportions of animals infected by trypanosome species in each sex, age, species, and season (critical value <0.05).

## Results

The overall frequency of trypanosome infection was 13.6% (39/286). Of the 146 sheep and 140 goats that were studied, 19.2% (28/146) of the sheep and 7.8% (11/140) of the goats were *Trypanosoma* positive (Table-1). The occurrence of trypanosome infection in males (16.4%) was higher than in females (12.5%). Adults had a lower prevalence (12.9%) than young (15%) (Table-1). However, these findings were more pronounced in sheep compared to goats (Table-2). Furthermore, no significant differences were observed between age (p=0.80) and sex (p=0.58) classes (Table-1).

**Table-1:** Overall prevalence of trypanosome infection in small ruminants.

Variables	Total number	Number of positive	Prevalence (%)	HP+	Comparison			

					χ^2^	p-value	df	S+
Sex								
Male	79	13	16.46	+	0.31	0.58	1	
Female	207	26	12.56					
Age								
Young	100	15	15	+	0.062	0.80	1	
Adult	186	24	12.90					
Species								
Sheep	146	28	19.17	+	5.13	0.024	1	+
Goat	140	11	8.57					
Season								
Dry	140	11	7.85	5.13	0.023	1	+
Rainy	146	28	19.18	+				

“Young” is ≤24 months; “Adult” is >24 months. HP+=Higher prevalence between different parameters, S+=Test significant for the Chi-square test

**Table-2:** Statistical analysis of *Trypanosoma* infection in sheep and goats of the Mongo County.

Variables	Goats		Sheep	
	
	χ^2^-test		χ^2^-test	
	
	n/N (%)	p-value	n/N (%)	p-value
Sex				
Male	2/27 7.41	1	11/52 21.15	0.876
Female	9/113 7.96		17/94 18.08	
Age				
Young	3/42 7.14	0.775	12/58 20.68	0.921
Adult	8/98 8.16		16/88 18.18	
Season				
Rainy	2/60 3.33	0.199	26/86 30.23	0.0013*
Dry	9/80 11.25		2/60 3.33	

*n* denotes the number of positive individual and N is total number of the analyzed individual

**Figure-1:**
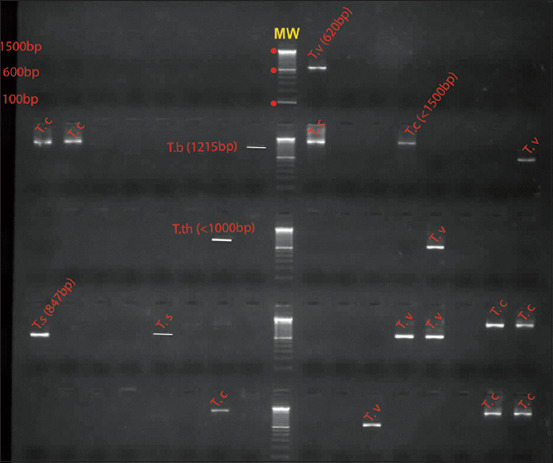
Gene amplification of trypanosomes using primers the ITS1, ITS2, ITS3, and ITS4 primers amplifying the 18S partial region for ITS1 and the 5.8S and 28S regions for ITS2. T.c=*Trypanosoma congolens*e; T.b=*Trypanosoma brucei*; T.v=*Trypanosoma vivax*; T.s=*Trypanosoma simiae*; T.th=*Trypanosoma theileri*; MW=Molecular weight.

Molecular analyses revealed that a total of six trypanosome species were found in small ruminants, more specifically, five *Trypanosoma* species in goats (*T. vivax, T. simiae, T. simiae* Tsavo*, T. congolense*, and *T. brucei*) and two in sheep (*T. simiae and T. theileri*) (Figure-1). Infection rates of *Trypanosoma* species varied between hosts (Table-3), though only for two species showed a significant difference between goats and sheep. Indeed*, T. simiae* was more prevalent (18.49%) in sheep (p=0.0061; d*f*=1), while *T. simiae* Tsavo was only found in goats with 5% of infection rate (p=0.022; d*f*=1).

**Table-3:** Parasites diversity found in small ruminants.

Trypanosoma species	Goats	Sheep	p-value χ^2^-test
*Trypanosoma vivax*	2.14% (3/140)	0 (0/146)	0.24
*Trypanosoma simiae*	5.71% (8/140)	18.49% (27/146)	0.0061*
*Trypanosoma simiae Tsavo*	5.00% (7/140)	0 (0/146)	0.022*
*Trypanosoma theileri*	0 (0/146)	1.37% (2/146)	0.502
*Trypanosoma brucei*	0.71% (1/140)	0 (0/146)	0.986
*Trypanosoma congolense*	0.71% (1/140)	0 (0/146)	0.986

This table reports the prevalence of each parasite identified and compares the prevalence between the goats and sheep. Statistical significance is indicated with an asterisk

Among the infected individuals, the frequency of mixed infections observed in goats was 54.5% (6/11), with various parasite associations. Only 3.57% (1/28) of sheep exhibited mixed infections (Table-4). The rates of coinfection in goats and sheep were statistically significant (p=0.013; d*f*=1; Chi-square=6.14). The coinfection *T. simiae*-*T. simiae* Tsavo was predominant in goats.

**Table-4:** Percentage of mixed infections detected in small ruminants.

Host species	Percentage of mixed infections	Parasite associated (n)
Goats	54.5% (6/11)	*Trypanosoma vivax*-*Trypanosoma simiae*-*Trypanosoma simiae Tsavo* (1)
		*Trypanosoma vivax*-*Trypanosoma simiae* (1)
		*Trypanosoma simae*-*Trypanosoma simiae Tsavo* (4)
Sheep	3.57% (1/28)	*Trypanosoma simiae*-*Trypanosoma theileri* (1)

*n*=Number of mixed infection found

Concerning the factors which could affect the infection level, the statistical analyses showed that two parameters could affect the infection rate: Species and season. Indeed, a significant difference was observed between the rainy and dry seasons (p=0.02; d*f*=1; Chi-square=5.13), with a higher infection level in the rainy season (Table-1). Table-2 shows that this difference was especially significant in sheep. The results also showed that the highest infection rate with trypanosome was recorded in sheep (18.4%), which was significantly higher (p=0.024; d*f*=1; Chi-square=5.13) than infection rate in goats (8.5%) (Table-1).

## Discussion

Trypanosomiasis in animals, particularly livestock, is a major impediment to livestock development in some rural areas of Africa affected by this disease. During these last few years, several studies reported many species circulating among farm animals [17-20]. Indeed, these studies about the disease burden of AAT in some livestock could invite a reevaluation of trypanosomiasis control strategy [21]. Few records exist on the effect of trypanosomosis in small ruminants in Africa, in particular Central Africa. Our study, which is the first in Gabon, assesses goat and sheep trypanosome infection prevalence and attempts to identify the diversity of species circulating in South Gabon.

In the present study, all identified species were already observed among small ruminants. Therefore, our study revealed that five *Trypanosoma* species infect goats (*T. vivax*, *T. simiae*, *T. simiae* Tsavo, *T. congolense*, and *T. brucei*) and two sheep (*T. simiae* and *T. theileri*). Infection rate ranged from 0.71 to 5.71% in goats and 1.37 to 18.49% among sheep, thus, our study revealed a higher rate of trypanosome infection in sheep than goats. These results are consistent with the previous studies that have shown sheep to be more frequently infected than goats [21,22]. This observation could be due the fact that goats are more refractory or resistant to trypanosome infections than sheep, as suggested by Ng’ayo *et al*. [21], which likely reflect the existence of some degree of trypanotolerance in goats [23]. Moreover, our results showed that sheep are more infected with *T. simiae* and *T. theileri*, while goats were only infected by three species (*T. simiae*, *T. simiae* Tsavo, and *T. vivax*). These observations contradict previous studies, which report that sheep are more infected with *T. congolense* and *T. brucei* than goats [24,25]. However, these two parasites were found only in goats but at a very low infection rate (0.71%).

The species richness of trypanosomes was higher in goats than sheep, with five species identified in goats (*T. vivax*, *T. simiae*, *T. simiae* Tsavo, *T. brucei*, and *T. congolense*) and only two in sheep (*T. simiae* and *T. theileri*). Infection level varied according to the host species for each parasite found. We observed that in goats, *T. simiae* and *T. simiae* Tsavo were the parasites with the highest frequencies, at 5.71% and 5%, respectively, and infection rate of *T. vivax* was 2.14%. However, for sheep, only two parasites were found: *T. simiae*, at 18.49% (the highest infection rate in this study), and *T. theileri* (1.34%). In Cameroon, *T. simiae* infection rate was 0.75% in goats and 0.37% in sheep [26]. The higher infection rate in small ruminants in Mongo County could be explained by the presence of various *T. simiae* reservoir animals. Indeed, this species, fatal in pigs, is predominant in wild pigs and can cause disease in small ruminants coexisting with wild pigs. T*. simiae* Tsavo, which causes mild disease in domestic pigs, has been found in warthogs [11].

*T. vivax, T. brucei*, and *T. congolense* were absent in sheep but present in goats with low infection rates. *T. congolense* and *T. brucei*, known to be important pathogens of cattle in Africa, were absent in sheep, as reported in the previous studies [21,25,27]. Our results support previous studies reporting the presence of these parasites in animals (dogs, cattle, and tsetse flies) at high prevalences [15,28-30].

Moreover, we observed mixed infections with two or three different *Trypanosoma* species, with the most common association being *T. simiae* and *T. simiae* Tsavo in goats. Our results support previous studies showing mixed infections of *Trypanosoma* species in livestock and the tsetse flies vectors of these parasites [21,31-33]. The mixed infections we observed in small ruminants could be explained by overexposure of animals to infected tsetse flies [31]. Thus, the presence of some *Trypanosoma* species could negatively impact human and animal health. Indeed, the presence of *Trypanosoma* species could affect the growth of farm animals [21]. Furthermore, a recent study reported cases of atypical human infections caused by animal trypanosomes species [34], including *T. congolense, T. brucei*, and *T. vivax* [34,35], that we found in this study. Thus, sheep and goats that are infected with a wide variety of trypanosomes could constitute a reservoir for these parasites, and the permanent contact of animals with humans could promote the crossing of the host barrier and lead to human infection with trypanosomes normally found in animals.

Finally, we also showed that host species and season influence trypanosome infection in small ruminants. Indeed, we observed that sheep were significantly more infected than the goats (p=0.028). Although sheep and goats are well known to be trypanotolerant [36], it has been shown that there is a difference in the infection rate between these two animal species for some pathogens [21,37,38]. Regarding season, we showed that trypanosome infection in small ruminants was higher in the rainy season, corroborating previous study [39] and possibly explained by an observed increase vector abundance during the rainy season [39,40]. Differences between age and sex classes did not reach statistical significance, though the higher prevalence we observed in females and young agrees with the previous studies [39]. Thus, we conclude that the factors we investigated likely have an influence on infection of small ruminants by trypanosomes.

## Conclusion

This study showed that small ruminants are reservoir animals of African trypanosomes. Small ruminants should be considered in trypanosomosis prophylaxis programs to prevent the spread of the disease. The study also suggests that wild animals could play a role in the spread of the disease in livestock. A study could also be undertaken to evaluate the impact of the disease on the productivity of these animals. However, further studies would be necessary to better characterize this *Trypanosoma* diversity, assess the risk to animal and human health, and understand the ecology of these parasites in Gabon.

## Authors’ Contributions

GDM conceived and designed the research. EJO and GDM conducted the sample collection. EJO, LBK, and TGNM carried out the molecular analysis. LB and EJO carried out the data analysis. GDM and LB wrote the manuscript. BK and JFM reviewed the manuscript. All authors read and approved the final manuscript.
